# p120 Catenin Is Required for the Stress Response in *Drosophila*

**DOI:** 10.1371/journal.pone.0083942

**Published:** 2013-12-12

**Authors:** Rhoda K. Stefanatos, Christin Bauer, Marcos Vidal

**Affiliations:** Drosophila Approaches to Cancer Laboratory, The Beatson Institute for Cancer Research, Glasgow, Scotland, United Kingdom; University of Bern, Switzerland

## Abstract

*p120ctn* is a ubiquitously expressed core component of cadherin junctions and essential for vertebrate development. Surprisingly, *Drosophila* p120ctn (dp120ctn) is dispensable for adherens junctions and development, which has discouraged *Drosophila* researchers from further pursuing the biological role of *dp120ctn*. Here we demonstrate that *dp120ctn* loss results in increased heat shock sensitivity and reduced animal lifespan, which are completely rescued by ectopic expression of a *dp120ctn-GFP* transgene. Transcriptomic analysis revealed multiple *relish*/*NF-κB* target genes differentially expressed upon loss of *dp120ctn*. Importantly, this aberrant gene expression was rescued by overexpression of dp120ctn-GFP or heterozygosity for *relish*. Our results uncover a novel role for *dp120ctn* in the regulation of animal stress response and immune signalling. This may represent an ancient role of *p120ctn* and can influence further studies in *Drosophila* and mammals.

## Introduction

*p120 catenin* (*p120ctn*) is the prototypic member of the p120ctn family in mammals and, together with *Armadillo Repeat gene deleted in Velco-Cardio-Facial syndrome* (*ARCVF*), *p0071* and *delta-catenin*, it binds the highly conserved juxta-membrane domain (JMD) of classic cadherins to promote adherens junction stability [1-3]. Catenins work in concert to link the intracellular domain of classical cadherins to the actin cytoskeleton to regulate cell-cell adhesion [3]. While the connection to the actin cytoskeleton via β-catenin and α-catenin is essential for cadherin-dependent adhesion, the role of p120ctn is accessory and regulates actin fibre contractility via regulation of the Rho GTPase [2,4-7]. In the absence of p120ctn, E-cadherin is endocytosed, resulting in weaker adhesion as junctions become more dynamic [6,8,9]. Furthermore, it has been shown that binding of p120ctn to the JMD of classic cadherins prevents modifications that target cadherin for degradation [10]. Vertebrate p120ctn has also been shown to play a role in Wnt signaling pathway [11] and repression of transcription through its association with the BTB domain-containing transcription factor Kaiso [12,13]. Not surprisingly, *p120ctn* is essential for development of vertebrate organs, including the mouse salivary gland, brain, kidney and mammary gland [14-17]. In the mouse adult intestine, loss of *p120ctn* leads to inflammation and increased adenoma formation [18,19]. Importantly, *p120ctn* has been shown to be down-regulated and mis-expressed in many types of cancer [20-27]. Due to its wide importance during vertebrate development, open questions remain about possible adhesion and Wnt signaling independent functions of p120ctn.

*Drosophila p120ctn* (*dp120ctn*) is the only member of the *p120ctn* family in flies and it localizes to the cytoplasm and adherens junctions [28,29]. In the cytoplasm, dp120ctn has been shown to bind Rho1. Whether this affects Rho1 activity is not known but there is evidence that dp120ctn preferentially binds to the GDP bound form of Rho1, suggesting it could have an inhibitory role in this context [30]. At the plasma membrane, dp120ctn binds to the conserved JMD domain of DE-cadherin. However unlike in vertebrates, this interaction does not affect DE-cadherin stability [28,29]. The generation of a null allele for *dp120ctn* demonstrated that this gene was dispensable for development, homeostasis and reproduction in *Drosophila* [29]. Indeed, re-expression of a mutant DE-cadherin that cannot bind dp120ctn rescued *shotgun/DE-cadherin* mutants, further confirming that dp120ctn binding to DE-cadherin is not required for development or homeostasis [28]. dp120ctn has so far not been observed in the nucleus in *Drosophila* and no homologous genes for Kaiso have been identified. Despite its dispensability for adhesion and development *dp120ctn* has been retained in the *Drosophila* genome and can be found in all invertebrates and vertebrates as a single or multi-gene family. This suggests that *dp120ctn* may have an important, yet unidentified, ancestral role [31,32]. 

In this study, we present evidence for a biological role of *Drosophila p120ctn*. Using a previously described null allele of *dp120ctn* (*p120* ^*308*^) we have found that *dp120ctn* null flies are hypersensitive to specific stressors and display overall reduced lifespan and differential expression of multiple immune response genes regulated by the transcription factor *relish/NF-κB*. Altogether, our results uncover a novel role for *p120ctn*, which may provide insights for future *Drosophila* and mammalian studies.

## Materials and Methods

### Fly husbandry

Flies were crossed and maintained on standard molasses media in incubators with under temperatures varying from 25°C to 29°C with a controlled 12hr-light:dark cycle. Fly stocks used in this study were kindly provided to us by our colleagues or obtained from the Bloomington Stock collection.

### Genotypes

*w* ^*-1118*^

*w* ^*-1118*^ *; p120* ^*308*^ **

*w* ^*-1118*^ *; ubiquitin-p120* ^*GFP*^

*w* ^*-1118*^ *; p120* ^*308*^ *; ubiquitin-p120* ^*GFP*^

*w* ^*-1118*^ *; p120* ^*308*^ *; relE20/+*

### Fly collection

Flies were collected using CO_2_ anaesthesia within 12 hours of eclosion and allowed to mate at 25°C for 2 days at a density of 20 flies per vial. Males and females were then separated and allowed to age at 25°C for a further 4 days.

### Heat shock assay

Flies were put into empty vials and placed in a water bath set at 37°C. Dead flies were counted at 15min intervals. 

### Paraquat assay

Flies were put into vials containing 5ml 1% agar and placed in a 25°C incubator for overnight starvation. Flies were then transferred to vials containing filter paper (Whatman®) soaked in 300μl of a 5% Sucrose solution with 20mM Paraquat and maintained at 25°C. Dead flies were counted at 12hr intervals. 

### H_2_0_2_ assay

Flies were starved overnight as for the Paraquat assay. Flies were then transferred to vials containing 5ml 1% agar plus a 5% Sucrose solution with 1% H_2_0_2_. Media was changed daily and dead flies were counted at 12hr intervals. 

### NaCl assay

After overnight starvation, flies were transferred to vials containing 5ml standard medium with 6% NaCl. Media was changed daily and dead flies were counted at 12hr intervals. 

### Analysis of stress test data

Survival graphs were created using Graphpad Prism software and log rank statistics applied. (100 male and 100 female flies were used for each experimental group – replicate experiments were performed from independent crosses). Animals were 6-7 days old for every stress test. Graphs presented depict results from one representative experiment. 

### Lifespan Analysis

Flies were collected within 24 hours of eclosion and mated for 48 hours. Flies were cultured at a density of 20 flies per vial at 25°C or 29°C in a controlled 12-hour light:dark cycle. Flies were transferred to new vials every 2-3 days and the number of dead flies was counted. Lifespan studies were performed with at least 100 flies per genotype. Survival curves were made using GraphPad Prism and log-rank tests were applied. 

### qRT-PCR

Total RNA was extracted and DNAase treated from triplicates of 10 adult females using the Qiagen RNAeasy kit. cDNA was synthesised using the High-Capacity cDNA reverse transcription kit (Applied Biosystems). cDNA was analysed in triplicate using an Applied Biosystems 7500 fast RT-qPCR machine. Primers pairs shown in Table S1 were used to measure mRNA levels of each target. Expression of the target genes was measured relative to that of *RpL32* (*rp49*). The primers used for the *Rpl32* housekeeping gene span introns. Melt curves were used to ensure the presence of a single product amplified with each primer pair. A series of 10-fold dilutions of an external standard was used in each run to produce a standard curve. Quanta SYBR green Master mix (lox ROX) was used following manufacturers instructions. Data were extracted and analysed using Applied Biosystems 7500 software version 2.0 and Prism GraphPad6 software. For each target mean fold change with standard error is presented.

### Microarray Analysis

Total RNA was extracted from triplicates of 10 adult female using the Qiagen RNAeasy kit. RNA was sent to the MIAMI microarray facility at the University of Manchester were it was used to hybridize *Drosophila* Affymetrix 2.0 chips. The cel files were normalised and analysed in Partek Genomics Suite Software by the Beatson Bioinformatics department. RMA normalisation and log2 transformation of the data was followed by the differential gene expression analysis using t-test. All p-values were corrected for multiple testing using Benjamini & Hochberg step up method that controls the false discovery rate. Data are deposited in the MIAMI microarray facility. 

Supplementary information is available online. 

## Results and Discussion

### *Drosophila p120ctn* mutants are sensitive to specific stressors

Knockdown of *dp120ctn* in *Drosophila* embryos using double stranded RNA (dsRNA) injection suggested an essential role for *dp120ctn* [30] and mild patterning defects have been reported in the developing retina of *dp120ctn* mutant flies [33]. Nevertheless, the generation of a *dp120ctn* null allele (*p120* ^*308*^) demonstrated that *dp120ctn* is not essential for development and homeostasis as *dp120ctn* mutant flies are viable and fertile [29]. Additionally, mutations in the JMD of DE-cadherin, which results in dp120ctn delocalisation, fully rescued *shotgun* (DE-cadherin) mutants [28]. We hypothesized that, while dispensable for development and adult tissue homeostasis, *dp120ctn* could be required in conditions of stress. To ensure that we observed only phenotypes associated with loss of *dp120ctn* we initially backcrossed flies carrying the null allele *p120* ^308^ with the control genotype *w* ^1118^ for ten generations to create our experimental line (*w* ^*1118*^ *; p120* ^*308*^). For all experiments we compared *w* ^1118^ (control) with *w* ^1118^ *; p120* ^308^ (*dp120ctn* mutants). We used males and females due to intrinsic differences in their ability to cope with stress.

Oxidative stress is characterized by an increase in the amount of reactive oxygen species (ROS) in a biological system and it has been linked to several pathologies. In order to induce oxidative stress in our system we used Paraquat and H_2_0_2_. Paraquat is an herbicide that is reduced within cells to increase ROS production, while H_2_0_2_ is a potent ROS itself. To examine the effects of oxidative stress on controls and *dp120ctn* mutants we fed 7-day old adults with a 5% sucrose medium containing either 20μM Paraquat or 1% H_2_0_2_. We then compared the survival of control and *dp120ctn* mutant animals in these conditions. We found no difference in survival associated with loss of *dp120ctn* in males or females on either Paraquat or H_2_0_2_ (Figure S1A-D).

Osmotic stress results in acute changes in water balance. To test whether *dp120ctn* mutants were more sensitive to increased osmotic stress we added sodium chloride (NaCl) at a concentration of 6% to standard *Drosophila* medium. We observed a mild but significant difference in survival between controls and *dp120ctn* mutant males (Figure S1E) but this was not mirrored in the case of females (Figure S1F). This suggested that, at this concentration of NaCl, *dp120ctn* males were sensitive to increased osmotic stress while females were not.

**Figure 1:**
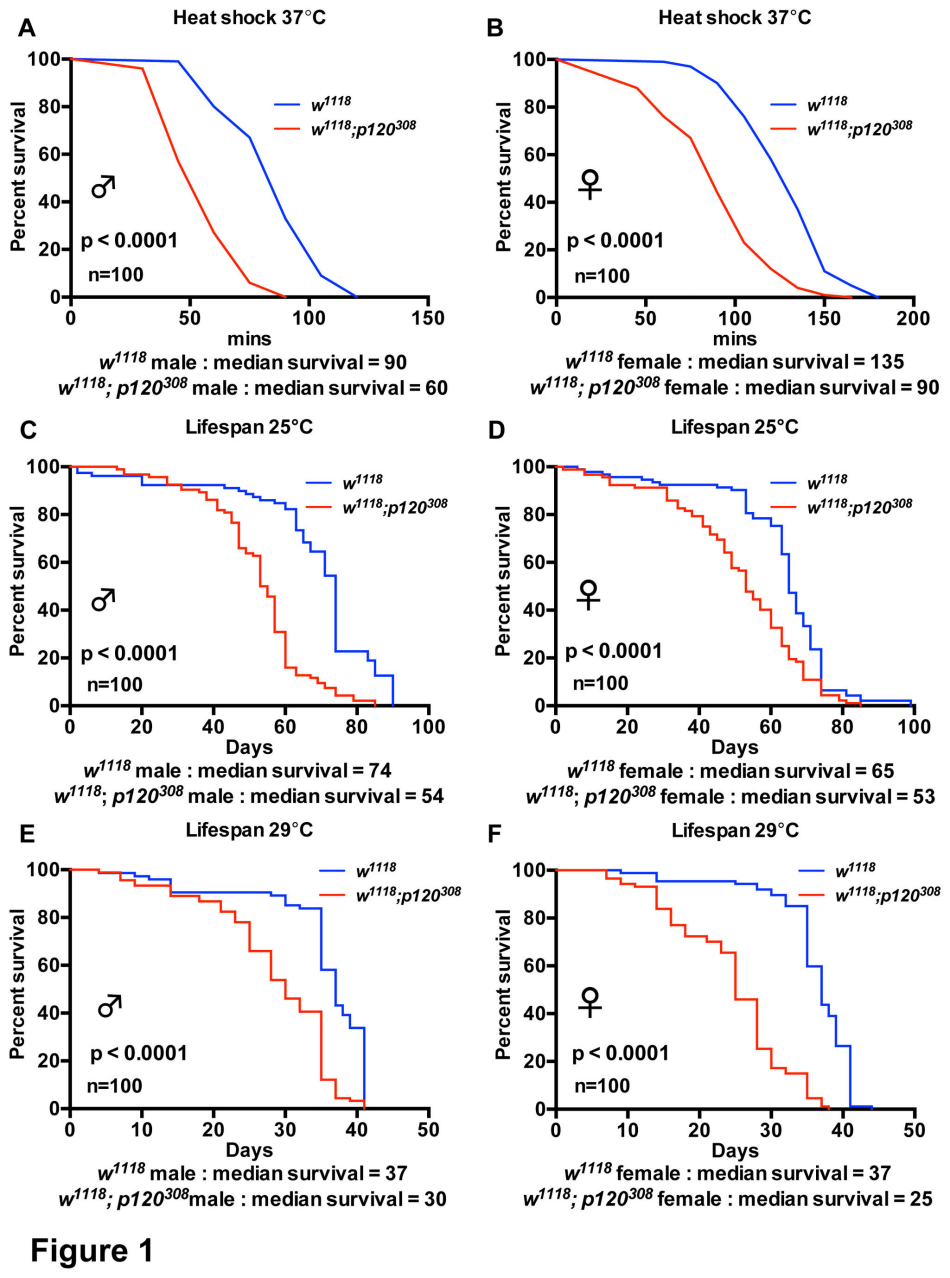
*dp120ctn* mutants are sensitive to heat shock and have a reduced median lifespan. (A-B) Survival of *w^1118^* and *w^1118^*; *p120^308^* males and females under heat shock conditions. Percent survival was plotted *versus* time in minutes under heat shock at 37°C. (C-F) Lifespan of *w^1118^* and *w^1118^*; *p120^308^* males and females at 25°C and 29°C. Survival was plotted as adult lifespan in days. Blue lines indicate *w^1118^* controls and red lines *w^1118^*; *p120^308^*. Each curve represents 100 flies. Median survival is presented for each cohort as well as *p* values generated using log-rank tests.

The organismal effect of heat shock has been heavily studied. Heat shock acts as a stress stimulus that induces transcription of specific genes, namely ‘Heat Shock proteins’. A number of these genes encode for protein chaperones that assist with protein folding and localization as well as with targeting of old or mis-folded proteins for degradation. Like most invertebrates, *Drosophilae* are unable to regulate their body temperature and, as such, prefer temperatures ranging from 18°C to 29°C. Therefore, to induce heat shock, we placed control and *dp120ctn* mutant flies at 37°C. We then compared survival of control males and females to *dp120ctn* mutant males and females. We found that both *dp120ctn* males and females were significantly more sensitive to heat shock (Figure 1A-B). This suggests that *dp120ctn* is functionally required under heat shock stress conditions.

Ageing is often characterized as the progressive deterioration of an animal. One consequence of the ageing process is the reduced ability to cope with environmental stress and this can be manifested as a reduced lifespan. We next measured the lifespan of control and *dp120ctn* mutant males and females at two different temperatures, 25°C and 29°C. At 25°C we observed a significant decrease in median lifespan in *dp120ctn* mutants, which was consistent in males and females (Figure 1C-D). Additionally, we could also observe a modest decrease in maximum lifespan (Figure 1C-D). We repeated the analysis at 29°C and observed the same outcome (Figure 1E-F). Together, these results suggest that the role of *dp120ctn* in the stress response is required beyond acute stress and affects animal lifespan. 

### Ubiquitous expression of dp120ctn-GFP rescues *dp120ctn* mutant stress sensitivity

**Figure 2:**
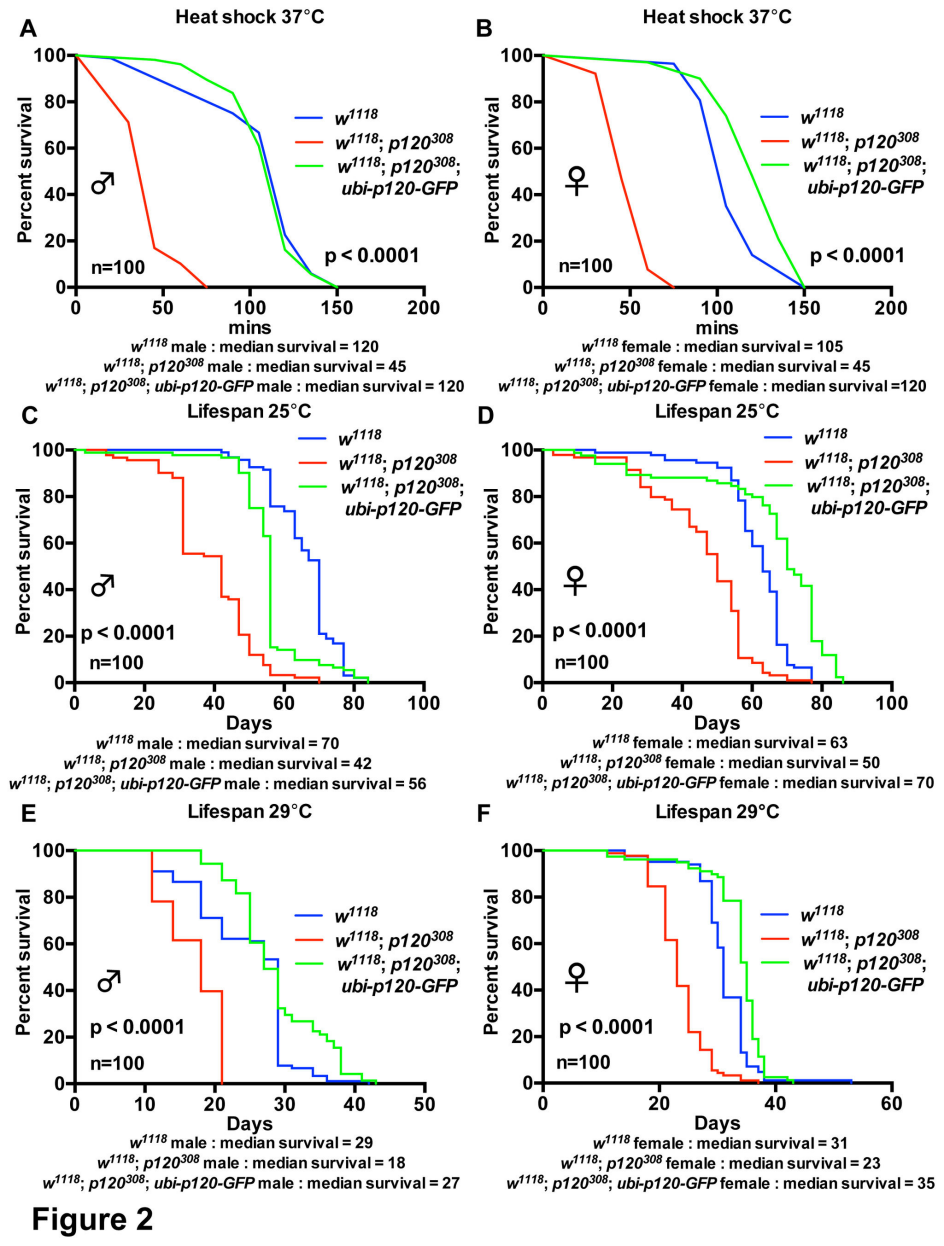
Ubiquitous Expression of dp120ctn-GFP rescues *dp120ctn* mutants. (A-B) Survival of *w^1118^*, *w^1118^*; *p120^308^* and *w^1118^*; *p120^308^*; *ubi-p120-GFP* males and females under heat shock conditions. Survival was plotted as adult survival in minutes under heat shock at 37°C. (C-F) Lifespan of *w^1118^* and *w^1118^*; *p120^308^* and *w^1118^*; *p120^308^*; *ubi-p120-GFP* males and females at 25°C and 29°C. Survival was plotted as adult lifespan in days. Blue lines indicate *w^1118^* controls, red lines *w^1118^*; *p120^308^* and green lines *w^1118^*; *p120^308^*; *ubi-p120ctn-GFP*. Each curve represents 100 flies. Median survival is presented for each cohort and *p* value generated using log-rank tests and refer to comparison between red lines (*w^1118^*; *p120^308^*) and green lines (*w^1118^*; *p120^308^*; *ubi-p120ctn-GFP*).

As we had backcrossed the *dp120ctn* null allele to the control strain we were confident that the stress sensitivity we had observed in *dp120ctn* mutant flies was as a result of the absence of *dp120ctn* rather than a genetic background effect. Nevertheless, to further confirm this we wanted to examine if we could rescue the mutant phenotypes through re-expression of *dp120ctn*. To do this we used a transgene expressing dp120ctn tagged with GFP under the control of the ubiquitin promoter (*ubiquitin-p120-GFP*) [29], which was backcrossed itself to the *w* ^1118^ strain for ten generations. Re-expression of dp120ctn-GFP completely rescued the sensitivity to heat shock and reduced lifespan observed in *dp120ctn* mutants (Figure 2). Therefore, all phenotypes of *dp120ctn* mutant animals are a specific result of *dp120ctn* loss.

### Transcriptomic analysis of *dp120ctn* mutants reveals differential gene expression profile

To gain insights into the mechanisms by which *dp120ctn* might be required in the response to specific stressors we performed transcriptomic analysis to get a global picture of gene expression in *dp120ctn* mutants. We isolated RNA from 7-day-old control and *dp120ctn* females as well as flies of the same age and genotype, which had been subjected to a 1-hour heat shock at 37°C. After purification, samples were hybridized to the Affymetrix GeneChip platform. After data collection, fold change and significance were calculated. A cut off of +/- 1.5 fold change was used to select genes for further analysis (see Materials and Methods).

**Figure 3:**
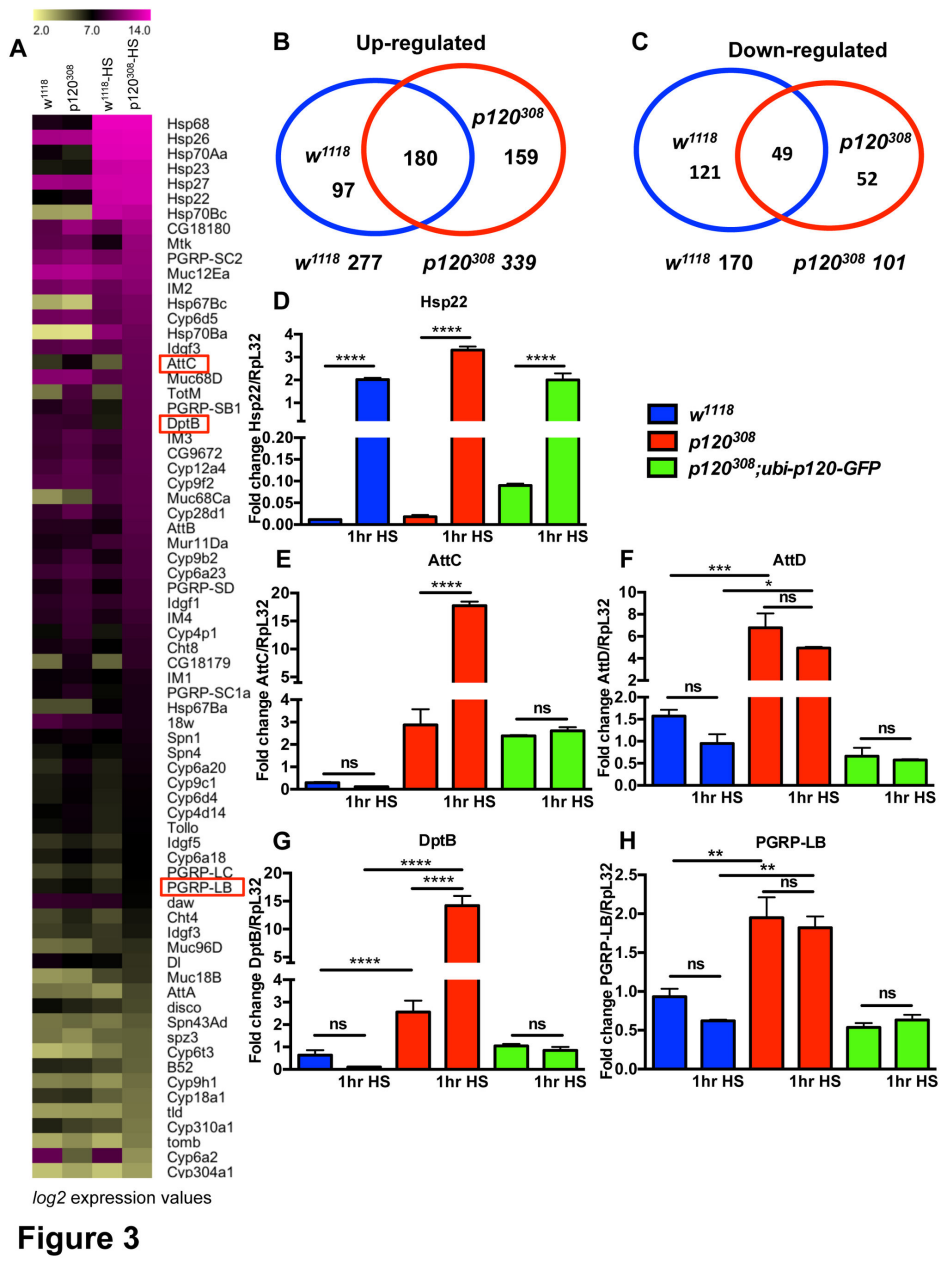
Transcriptomic analysis of *dp120ctn* mutants reveals differential gene expression. (A) Averaged expression profile (*log2* expression values) of selected genes in *w^1118^* and *w^1118^*; *p120^308^* flies before (*w^1118^*, *w^1118^*; *p120^308^*) and after 1hr of heat shock (*w^1118^*-HS, *w^1118^*; *p120^308^*-HS). Some of the genes selected for qPCR analysis are boxed in red. (B-C) Diagrams showing the number of genes up-regulated and down-regulated in *w^1118^* and *w^1118^*; *p120^308^* flies in response to 1hr heat shock, and their overlap. Note that there were more genes up-regulated upon heat shock in the mutants (D-H). qPCR analysis of selected targets in *w^1118^*, *w^1118^*; *p120^308^* and *w^1118^*; *p120^308^*; *ubi-p120-GFP* flies before and after 1hr heat shock (one-way ANOVA with Bonferroni post test correction, ns: non-significant, * p<0.05; **p<0.005, ***p<0.001****p<0.0001). Note that the classic stress response gene *hsp22* was similarly up-regulated in all genotypes. Instead, genes previously associated with the IMD immune response such as AttC and DptB were up-regulated specifically in the mutants upon stress, and these together with other immune genes such as AttD and PGRP-LB were constitutively up-regulated in the mutations without stress challenge. (All error bars denote standard error of the mean).

 We first compared how each genotype responded to the 1-hour heat shock (Figure 3A, Figure S2). We could appreciate that, although there were many genes equally up-regulated and down-regulated in response to heat shock, there were also many genes that behaved distinctly in each genotype. We found that, overall, more genes were induced than repressed in both control and *dp120ctn* mutant flies after heat shock (Figure 3B-C). *dp120ctn* mutant flies showed approximately 60 more genes up-regulated than control flies (Figure 3B). Additionally, only around half the genes induced in control and *dp120ctn* flies were common between both groups. A significant amount of genes were also down-regulated in response to heat shock but, in this case, control flies down-regulated around 70 more genes than *dp120ctn* mutant flies and only around half the genes down-regulated in control were also down-regulated in *dp120ctn* mutant flies (Figure 3C). From this first assessment we could conclude that, indeed, there was a transcriptional response to heat shock in *dp120ctn* mutant flies, however it did not completely match that of control flies. 

We also wanted to ask whether *dp120ctn* mutant flies had a distinct transcriptional profile from control flies without any stress stimulus. We found around 700 genes were differentially expressed in *dp120ctn* mutant flies (Figure 3A). A careful examination of the data indicated that several of the differentially regulated genes were previously associated with the response to pathogenic infection. Two previous studies from *De Gregorio et al* had investigated the transcriptomic response to immune challenge [34,35]. These studies identified around 400 genes, which they named *Drosophila* immune-regulated genes (DIRGs), that were regulated in response to infection [34]. They went on to extend this by defining which DIRGs were targets of the Immune deficiency pathway (IMD) or the Toll signaling pathway. IMD and Toll represent the two branches of the innate immune response in *Drosophila* and are each upstream of the NF-κB like transcription factors *relish* (IMD) and *Dorsal* (Toll), respectively [35,36]. In their studies *De Gregorio et al* described around 34 targets, which were differentially regulated in response to infection downstream of the *relish* transcription factor. We found that 20 of these *relish* dependent DIRGs were differentially regulated in *dp120ctn* mutant flies. To confirm these hits we performed quantitative RT-PCR (qRT-PCR) on samples from (i) control, (ii) *dp120ctn* mutant and (iii) *dp120ctn-GFP* rescue unchallenged flies as well as from flies of the same genotypes that had been subjected to a 1 hour heat shock. 

We first examined the heat shock response gene, *Hsp22*, which is induced readily upon heat shock. We found that all flies subjected to heat shock showed elevated levels of *Hsp22* mRNA, irrespective of the genotype (Figure 3D). We next measured the expression levels of three antimicrobial peptides (AMPs), *attacin C* (*AttC*), attacin D (*AttD*) and *Diptericin B* (*DptB*), which are induced in response to infection through the IMD pathway [35,36] (Figure 3E-G). In all three cases, expression of these genes in *dp120ctn* mutants was significantly increased even before heat shock treatment (Figure 3E-G). After heat shock *AttC* and *DptB* were significantly up regulated specifically in *dp120ctn* mutant flies (Figure 3E,G). Furthermore, these effects were completely abolished in *dp120ctn*-*GFP* rescue flies (Figure 3E-G). We also measured the mRNA levels of another DIRG, the peptidoglycan recognition protein LB (PGRP-LB) in the same samples and found that *dp120ctn* mutant flies expressed higher levels of this gene before heat shock and that its mis-regulation was rescued in *dp120ctn-GFP* flies (Figure 3H). These data strongly support a role for *dp120ctn* in the negative regulation of immune response genes, as *dp120ctn* mutants express genes normally induced in the IMD/*relish* dependent transcriptional immune response. This is in accordance with evidence from previous reports that loss of *p120ctn* in the murine epidermis results in acute inflammation due to constitutive NF-κB activation [37]. 

**Figure 4:**
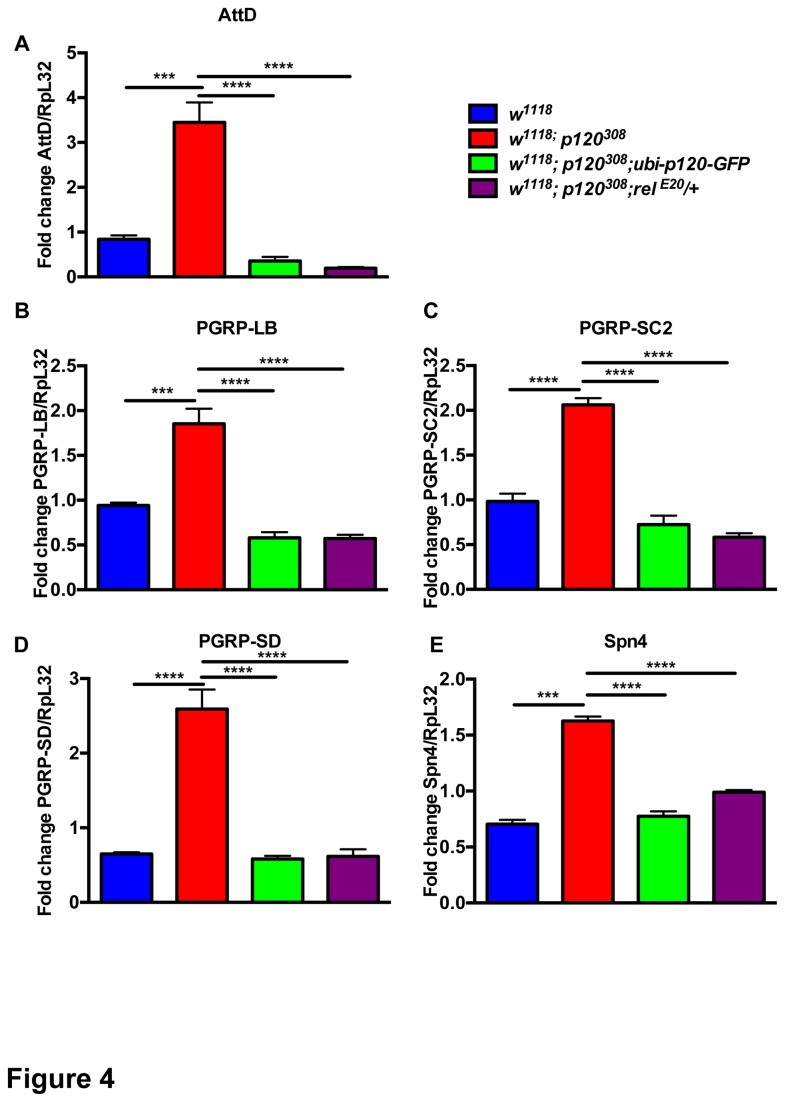
Ubiquitous expression of dp120ctn-GFP or relish heterozygosity rescues aberrant gene expression in *dp120ctn* mutant flies. (A-E) qPCR analysis of the selected genes in (i) *w^1118^*, (ii) *w^1118^*; *p120^308^*, (iii) *w^1118^*; *p120^308^*; *ubi-p120-GFP* and (iv) *w^1118^*; *p120^308^*; *relE20/*+ flies (one-way ANOVA with Bonferroni posttest correction, ***p<0.001****p<0.0001). Note that restoring p120ctn expression or halving the genetic dose of relish rescued the deregulated expression of these genes in *dp120ctn* mutants. (All error bars denote standard error of the mean).

We hypothesized that this NF-κB activation could also be occurring in *dp120ctn* mutant flies. To test this further we combined *dp120ctn* mutant flies with heterozygosity for a null allele of *relish* (*relE20*) (*w* ^*1118*^ *; p120* ^*308*^ *; relE20/+*) and measured levels of *relish* regulated DIRGs (*AttD, PGRP-LB*, *PGRP-SC2*, *PGRP-SD* and *Spn4*) (Figure 4A-E). Remarkably, halving the dose of *relish* or expression of *dp120-GFP* rescued the expression levels of these key targets (Figure 4A-E). This suggests that *dp120ctn* is required to negatively regulate *relish* dependent transcription. Many immune genes participate in the stress response [38], and their deregulation in *dp120ctn* mutants may cause the hypersensitivity to stress. Nevertheless, the fact that these genes were not regulated by stress in the control genotypes argues against this possibility. Alternatively, the sensitivity to stress may be indirectly linked to reduced animal fitness due to unnecessary expression of immune genes.

Studies in the murine epidermis, intestine and oral and oesophageal tract [18,19,37,39] have reported inflammation and increased immune cell recruitment upon loss of *p120ctn*. The results presented here suggest a conserved role for *p120ctn* in the regulation of immune signalling in *Drosophila* that may be independent of its role in adhesion. This could be a direct or indirect regulation. A recent study in endothelial cells described a role for p120ctn in the negative regulation of Toll-like receptor (TLR) 4 signalling through inhibition of the interaction between TLR-4 and the universal adaptor MYD88 [40]. In this context, our data suggest that dp120ctn could regulate the strength of an immune response through direct interactions with canonical members of IMD signalling in *Drosophila*. 

The ancestral role of the *p120ctn* family of proteins may have been to control the level of the immune response, while its roles in regulating cadherin turnover and Wnt signalling may have appeared during the evolution of the vertebrate lineages. Since the original roles of proteins are usually fixed in evolution, it is likely that mammalian *p120ctn* also participates in the stress response. 

## Supporting Information

Table S1 **Primers pairs used in this study.** Primer pairs used for qRT-PCR are shown.
(DOCX)

Figure S1 **p120ctn mutants are not sensitive to oxidative or salt stress.** (A-B) Survival of *w^1118^* and *w^1118^*; *p120^308^* males and females treated with 20uM Paraquat. Data are plotted as adult survival in hours. (C-D) Survival of *w^1118^* and *w^1118^*; *p120^308^* males and females treated with 1% H_2_0_2_. Data is plotted as adult survival in hours. (E-F) Survival of *w^1118^* and *w^1118^*; *p120^308^* males and females treated with 6% NaCl. Data is plotted as adult survival in hours. Blue lines indicate *w^1118^* controls and red lines *w^1118^*; *p120^308^*. Each curve represents 100 flies. Median survival is presented for each cohort and *p* value generated using log-rank tests.
(TIF)

Figure S2 **Transcriptomic analysis of *w^1118^* and *w^1118^*; *p120^308^* flies.** (A) Expression profile (*log2* expression values) of selected genes in *w^1118^* and *w^1118^*; *p120^308^* flies before (*w^1118^*, *w^1118^*; *p120^308^*) and after 1hr of heat shock (*w^1118^-*HS, *w^1118^*; *p120^308^*-HS). Each of the biological triplicates is shown.
(TIF)
