# Supplementary material for: p120 Catenin Is Required for the Stress Response in Drosophila
Source: PLoS One. 2013 Dec 12;8(12):e83942. doi: 10.1371/journal.pone.0083942 (PMC3861524; doi:10.1371/journal.pone.0083942)
Supplement: Table S1 — Primers pairs used in this study. Primer pairs used for qRT-PCR are shown. (DOCX) [file pone.0083942.s001.docx]

| Primer Name | Seq (5’-3’) |
| --- | --- |
| RpL32 F | AGGCCCAAGATCGTGAAGAA |
| RpL32 A | TGTGCACCAGGAACTTCTTGAA |
| hsp22F1 | CCGTCAACAAGGATGGCTAC |
| hsp22R1 | CTCTGATTTTCCCTCCACCA |
| AttAF1 | ATGCTCGTTTGGATCTGACC |
| AttAR1 | AAAGTTCCGCCAGTTGTGAC |
| AttBF1 | GTCATGGTGCCTCTTTGACC |
| AttBR1 | CCAGATTGTGTCTGCCATTG |
| AttCF1 | CAACGGTGTTCACAATCTGG |
| AttCR1 | GGGAGTAGTCCAGTGCAGCTT |
| AttDF1 | AGTGGGGGTCACTAGGGTTC |
| AttDR1 | GTGGCGTTGAGGTTGAGATT |
| DptBF1 | GGATTCGATCTGAGCCTCAAC |
| DptBR1 | ATAGGGTCCACCAAGGTGCT |
| PGRP-LB-F1 | TCTCCAATCTCCGATCAGCA |
| PGRP-LB-R1 | GCGATGGCATGATTTACACC |
| PGRP-LC-F1 | CCGAAGCGGAGGATTATACG |
| PGRP-LC-R1 | GGCGACTGATCACCGTTAGA |
| PGRP-SB1-F1 | AGACGATGCCAATGCTCTTG |
| PGRP-SB1-R1 | ACTTTATCGTGGCCGGTGAT |
| PGRP-SC2F1 | CGTGACCATCATCTCCAAGTC |
| PGRP-SC2R1 | GTAGTTTCCAGCGGTGTGGT |
| PGRP-SD-F1 | CAGCTGGTGGAGGGCTATAA |
| PGRP-SD-R1 | AGTTGGGCCACTGCTGTATC |
| Spn4F1 | TCTGGGCGGGTTAAGTGTTT |
| Spn4R1 | CTTGTGCCGGCTGATGTATT |

Supplementary Table S1
