# Supplementary figures and images for: p120 Catenin Is Required for the Stress Response in Drosophila
Source: PLoS One. 2013 Dec 12;8(12):e83942. doi: 10.1371/journal.pone.0083942 (PMC3861524; doi:10.1371/journal.pone.0083942)

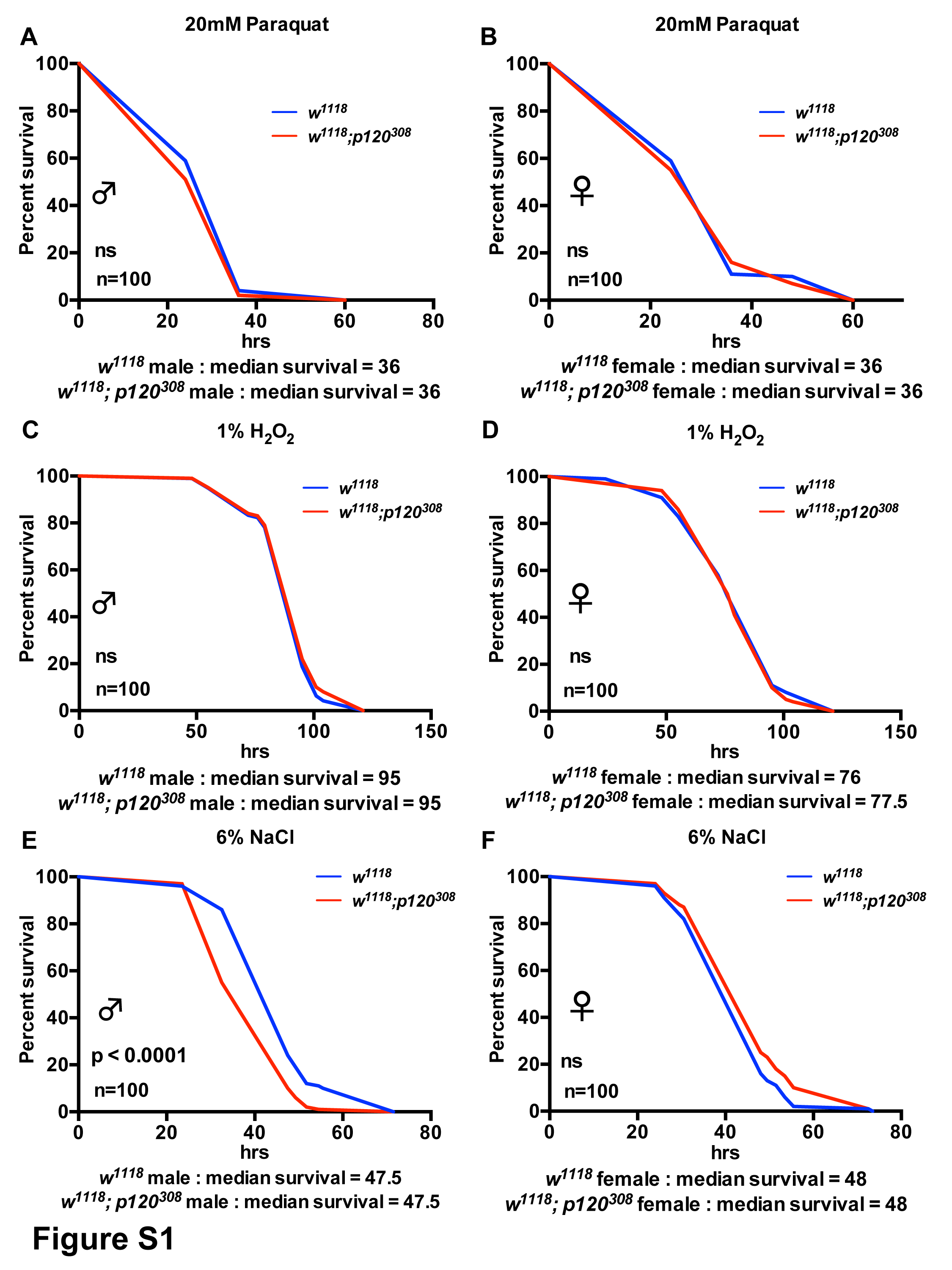

Supplement: Figure S1 — p120ctn mutants are not sensitive to oxidative or salt stress. (A-B) Survival of w1118 and w1118; p120308 males and females treated with 20uM Paraquat. Data are plotted as adult survival in hours. (C-D) Survival of w1118 and w1118; p120308 males and females treated with 1% H202. Data is plotted as adult survival in hours. (E-F) Survival of w1118 and w1118; p120308 males and females treated with 6% NaCl. Data is plotted as adult survival in hours. Blue lines indicate w1118 controls and red lines w1118; p120308. Each curve represents 100 flies. Median survival is presented for each cohort and p value generated using log-rank tests. (TIF) [file pone.0083942.s002.tif]

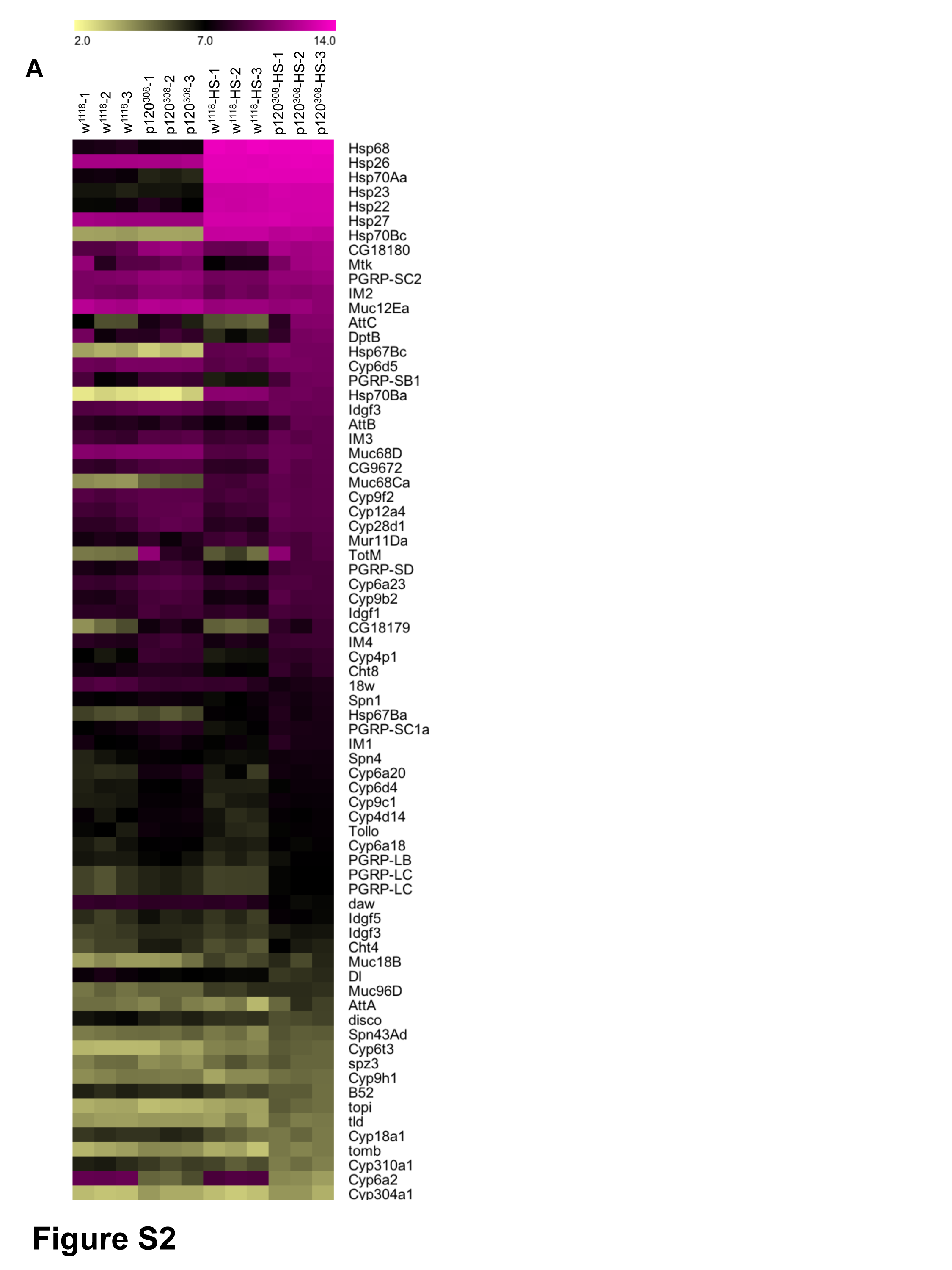

Supplement: Figure S2 — Transcriptomic analysis of w1118 and w1118; p120308 flies. (A) Expression profile (log2 expression values) of selected genes in w1118 and w1118; p120308 flies before (w1118, w1118; p120308) and after 1hr of heat shock (w1118-HS, w1118; p120308-HS). Each of the biological triplicates is shown. (TIF) [file pone.0083942.s003.tif]
